# Thermal and Stability Outcomes of Different Osteotomy Techniques and Implant Macrogeometries in Type IV Bone: An In Vitro Study

**DOI:** 10.3390/bioengineering12111155

**Published:** 2025-10-24

**Authors:** F. Gülfeşan Çanakçi, Merve Çakır, Gül Merve Yalcin-Ülker, Gonca Duygu

**Affiliations:** 1 Department of Oral and Maxillofacial Surgery, Faculty of Dentistry, Trakya University, 22030 Edirne, Türkiye; 2 Department of Oral and Maxillofacial Surgery, Faculty of Dentistry, Okan University, 34959 Istanbul, Türkiye; dtmervecakir@gmail.com (M.Ç.); gmerveyalcin@gmail.com (G.M.Y.-Ü.); 3 Department of Oral and Maxillofacial Surgery, Faculty of Dentistry, Namik Kemal University, 59030 Tekirdağ, Türkiye; duygu_gonca@hotmail.com

**Keywords:** dental implants, type IV bone, osseodensification, conventional osteotomy, low-speed osteotomy, osseoshaper, insertion torque, implant stability quotient, thermal changes, implant macrogeometry

## Abstract

Achieving reliable implant stability in type IV (D4) bone remains a clinical challenge due to its thin cortical plates and low trabecular density, which increase the risk of early failure. Novel osteotomy techniques such as osseodensification and the OsseoShaper have been proposed, yet their effects when combined with different implant macrogeometries are not fully understood. This in vitro study evaluated forty osteotomies in standardized polyurethane foam blocks simulating D4 bone density. Three site preparation protocols—conventional osteotomy, osseodensification, and OsseoShaper—were combined with two implant macrogeometries (parallel-walled conical and tri-oval tapered designs). Insertion torque (IT) was measured using a calibrated digital torque meter, and implant stability was assessed by resonance frequency analysis (ISQ). Intraoperative thermal changes were monitored with infrared thermography under constant irrigation. Statistically significant differences were observed among groups (*p* < 0.05). Osseodensification with parallel-walled implants achieved the highest stability, whereas osseodensification with tri-oval implants showed the lowest. Although osseodensification produced the greatest thermal increase, all values remained below the 47 °C osteonecrosis threshold. Within the study’s limitations, both the osteotomy technique and implant macrogeometry significantly affected stability and thermal outcomes, with osseodensification plus parallel-walled implants providing the most predictable performance in D4 bone.

## 1. Introduction

Primary implant stability is a key determinant of osseointegration and long-term success in implant dentistry. However, achieving reliable stability in type IV (D4) bone remains a challenge for clinicians. D4 bone, characterized by thin cortical plates and low trabecular density, is associated with higher early failure rates and difficulties in achieving stable fixation compared with denser bone types [1]. One of the most cited clinical reports, by Jaffin and Berman, showed significantly higher implant failure rates in D4 bone, highlighting bone quality as a critical factor in treatment outcomes [2]. To standardize preclinical testing, rigid polyurethane foam blocks are often used because they provide reproducible mechanical properties and are widely accepted for evaluating implant site preparation techniques across different densities [3].

Conventional osteotomy (CO) remains the most common approach, but it involves the removal of native bone. To overcome this limitation, alternative methods have been developed to preserve or densify trabecular structures. One of these is osseodensification (OD), which uses burs in counterclockwise rotation to compact bone, thereby increasing local density and stability [4,5,6]. Another approach, the OsseoShaper (OS), prepares sites at low speed (~50 rpm) without irrigation, aiming to minimize thermal changes and mechanical trauma while preserving vital bone [7,8]. Clinical and preclinical reports describe its biologically friendly rationale and the tri-oval implant macrogeometry designed to optimize load distribution [7,9,10].

Beyond mechanical stability, thermal safety is another critical consideration. It is well established that exposure to 47 °C for about 1 min increases the risk of osteonecrosis [11]. Thermal changes depend on multiple factors, including drill geometry, operating speed, irrigation, and bone quality [11,12]. Recent investigations have examined how these factors interact in subtractive, single-step, and low-speed approaches, including their behavior in D4 bone [13,14].

However, findings regarding the mechanical and biological advantages of these protocols remain inconsistent. Bandela et al. [15] used an ex vivo sheep iliac model to show that OD significantly improved insertion torque, implant stability quotient, and reverse torque compared with CO. Similarly, Orth et al. [16] reported higher stability for OD than for bone expansion systems, particularly in D4 bone and narrow implants. By contrast, Politi et al. [17] observed that at certain time points, implants placed with CO achieved greater stability than OD, suggesting that the benefits of OD may not be universal. Saqr et al. [18] reported in a randomized clinical trial that OD promoted ridge expansion and greater primary stability, but these advantages were limited at apical and deeper measurement points.

Given these considerations, it is important to clarify how osteotomy protocols and implant macrogeometries interact in low-density conditions [19]. While several studies have examined them separately, limited evidence addresses their combined influence in D4 bone. This is clinically relevant because the poorest bone quality often coincides with challenging anatomical conditions, where both surgical protocol and implant design may determine treatment success. Therefore, this study compared three osteotomy techniques (CO, OD, and OS) combined with two implant macrogeometries: a parallel-walled conical design and a tri-oval tapered design. The null hypothesis was that no statistically significant differences would be found among groups in terms of insertion torque (IT), implant stability quotient (ISQ), and thermal changes (ΔT). Findings from this work are expected to provide clinicians with practical insights for optimizing implant placement strategies in low-density bone conditions.

## 2. Materials and Methods

### 2.1. Study Design

Two implant macrogeometries were tested: NobelParallel Conical Connection (NPCC) implants (4.3 mm diameter, 11.5 mm length; Nobel Biocare, Gothenburg, Sweden) and Nobel Biocare N1 (tri-oval tapered) (N1) implants (4.0 mm diameter, 11 mm length; Nobel Biocare, Gothenburg, Sweden). Each implant type was placed following three different site-preparation techniques: conventional osteotomy (CO), osseodensification (OD), and OsseoShaper (OS). Since the study was performed in vitro using rigid polyurethane foam blocks, ethical approval was not required. Generative artificial intelligence (GenAI) was used only for figure preparation and for superficial text editing. No GenAI tools were used in the study design, data collection, analysis, or interpretation.

### 2.2. Bone Model

This in vitro study used standardized rigid polyurethane foam blocks (Sawbones, Pacific Research Laboratories, Vashon, WA, USA; Ref. No: 1522-01) with a density of 10 pounds per cubic foot (PCF). The blocks had a density of 0.16 g/cm^3^, a compressive yield strength of approximately 2.2 MPa, and a compressive modulus of about 58 MPa. This model has been widely used to represent low-density type IV bone in implant stability and thermal studies [20,21]. According to ASTM standards (ASTM F1839-08), it is suitable for testing endosseous implants and surgical instruments [22].

### 2.3. Study Groups

**Figure 1:**
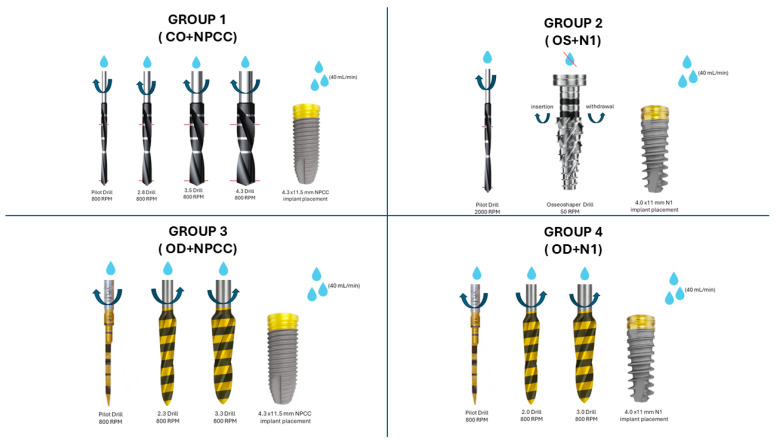
Schematic representation of the osteotomy protocols tested. CO was performed with sequential drills under irrigation; OS at prepared sites at low speed without irrigation according to the manufacturer’s instructions; and OD was carried out with counterclockwise Densah^®^ burs under irrigation. These protocols were combined with either parallel-walled or tri-oval designs.

A total of 40 osteotomies were prepared in the rigid polyurethane foam blocks. Power analysis was performed using G*Power (v3.1.9.7, Heinrich Heine University, Düsseldorf, Germany). Based on the study by Seo et al. [23], a sample size of 10 osteotomies per group was determined to provide 90% power to detect significant differences with an effect size of f = 0.73 and a significance level of α = 0.05. Randomization was performed using a computer-generated list to ensure an equal allocation of 10 specimens per group. All procedures were performed by the same operator to minimize inter-operator variability. The study groups are summarized in Table 1, and schematic osteotomy protocols are illustrated in Figure 1. Representative images of the osteotomy site preparations are provided in Supplementary Figure S1.

### 2.4. Implant Placement and Primary Stability Assessment

After site preparation, implants were inserted according to group allocation.

Insertion Torque (IT): Peak IT values (N·cm) were measured during implant placement using the OsseoSet™ 300 surgical unit (Nobel Biocare, Gothenburg, Sweden; manufactured by W&H Dentalwerk Bürmoos GmbH, Bürmoos, Austria), calibrated according to the manufacturer’s instructions prior to the study.

Resonance Frequency Analysis (RFA): Implant Stability Quotient (ISQ) values were obtained with a Penguin RFA device (Penguin Instruments, Gothenburg, Sweden). Multipegs were hand-tightened according to the implant system: Ref. No. 55048 for NPCC implants and Ref. No. 55126 for N1 implants. Readings were taken in buccolingual and mesiodistal directions, and the mean of two measurements was used for statistical analysis. Representative images of the RFA measurement setup are provided in Supplementary Figure S2.

### 2.5. Thermal Measurements

All osteotomies were performed at room temperature (21 ± 1 °C). Intraoperative thermal changes (ΔT) were recorded with an infrared thermal camera (UNI-T UTI720E, UNI-T, Dongguan, China) positioned 25 cm from the osteotomy site. Representative thermal images captured during osteotomy are provided in Supplementary Figure S3. The camera was fixed in place using the original foam holder to maintain constant alignment. The device specifications were as follows: infrared resolution: 256 × 192 pixels, field of view: 56° × 42°, spectral range: 8–14 μm, thermal sensitivity (NETD): ≤50 mK, accuracy: ±2 °C or ±2%, and image acquisition frequency: 25 Hz. The emissivity was standardized to 0.95 for polyurethane materials. The device was factory-calibrated before the experiment.

**Figure 2:**
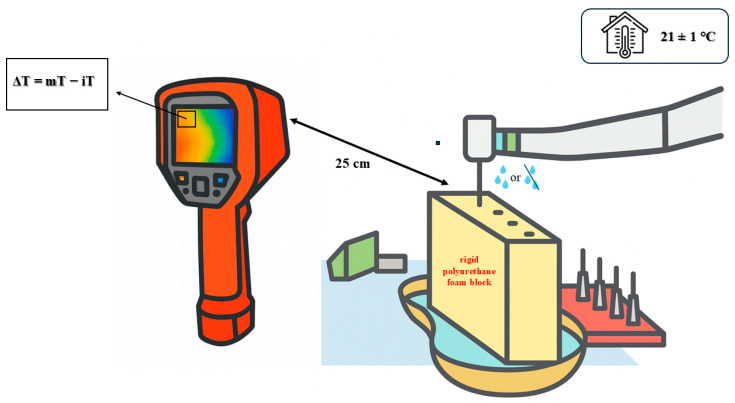
Illustrative schematic of the experimental setup used for intraoperative thermal measurements. The infrared thermal camera (UNI-T UTI720E) was positioned 25 cm from the osteotomy site and fixed in a foam holder to ensure constant alignment. The alveolar crest region was selected as the single-point measurement zone (crosshair).

A fixed crosshair was placed at the alveolar crest in the thermal video display, and single-point temperature values were recorded. The baseline temperature (iT) was defined as the crosshair value during the 1 s immediately before osteotomy, while the maximum temperature (mT) was defined as the highest crosshair value during drilling. ΔT was calculated as mT − iT. Osteotomies were spaced at least 35 mm apart, and each block was allowed to return to baseline temperature before the next drilling session to avoid cumulative heating. Figure 2 illustrates the experimental setup used for thermal measurement during osteotomy procedures.

### 2.6. Statistical Analysis

Data were analyzed with SPSS software (version 29.0; IBM Corp., Armonk, NY, USA). The Shapiro–Wilk test indicated non-normal distributions, and non-parametric tests were therefore used. Intergroup comparisons were made with the Kruskal–Wallis test, followed by Dunn–Bonferroni post hoc tests when significant. Results are reported as medians with ranges (minimum–maximum). Statistical significance was set at *p* < 0.05, with a 95% confidence interval. All measurements were performed by the same operator, and intraobserver consistency was verified by repeating 10% of randomly selected measurements, which showed high reproducibility.

## 3. Results

### 3.1. Insertion Torque (IT)

**Figure 3:**
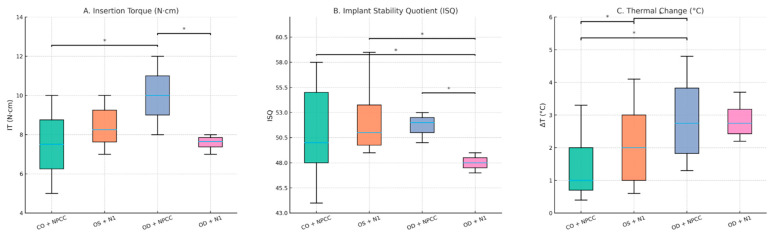
Box plots of the outcome variables across the four study groups. (**A**) Insertion torque (IT), (**B**) implant stability quotient (ISQ), and (**C**) thermal change (ΔT). Boxes represent interquartile ranges with horizontal lines indicating medians; whiskers denote minimum and maximum values. Significant differences between groups are indicated (*p <* 0.05, Kruskal–Wallis with Dunn–Bonferroni test). OD + NPCC implants demonstrated the highest torque and stability but also the greatest ΔT, while OD + N1 showed the lowest stability values. * *p* < 0.05 indicates statistically significant differences between groups.

Significant differences in IT were observed among groups (Kruskal–Wallis, *p* = 0.001). Median values are summarized in Table 2, and distribution patterns are illustrated in Figure 3A. Post hoc analysis revealed that Group 3 (OD + NPCC, 10.0 N·cm) exhibited significantly higher IT compared with Group 1 (CO + NPCC, *p* = 0.040) and Group 4 (OD + N1, *p* = 0.002) (Table 3).

### 3.2. Resonance Frequency Analysis (ISQ)

ISQ values also differed significantly among groups (*p* = 0.001). Median values are provided in Table 2, and distributions are shown in Figure 3B. Group 4 (OD + N1, 48.75) demonstrated significantly lower ISQ compared with Group 1 (CO + NPCC, *p* = 0.040), Group 2 (OS + N1, *p* = 0.002), and Group 3 (OD + NPCC, *p* = 0.001) (Table 3).

### 3.3. Thermal Changes (ΔT)

Thermal changes varied significantly across groups (*p* = 0.001). Median values are presented in Table 2, with distributions illustrated in Figure 3C. Group 1 (CO + NPCC, 0.70 °C) demonstrated the lowest ΔT, whereas Group 3 (OD + NPCC, 3.35 °C) produced the highest. Post hoc analysis confirmed significant differences between Group 3 and Group 1 (*p* = 0.004), as well as between Group 3 and Group 2 (OS + N1, *p* = 0.010) (Table 3).

## 4. Discussion

This in vitro study examined how three osteotomy protocols (CO, OD, and OS) combined with two implant macrogeometries influenced insertion torque, implant stability quotient, and thermal changes in type IV bone. The null hypothesis, which stated that no significant differences would be found among groups, was rejected, as all outcomes varied significantly depending on the protocol and implant design.

Our findings demonstrated that OD combined with the NPCC implant achieved the highest primary stability, whereas OD combined with the N1 (tri-oval design) produced the lowest stability. These results are in line with previous studies confirming that OD can enhance stability through trabecular bone compaction [24,25,26,27,28]. However, the lack of benefit observed with the tri-oval implant design may reflect a biomechanical mismatch, as densified cylindrical osteotomy walls do not fully engage with non-cylindrical macrogeometries. Similar outcomes were reported by Fabbri et al. [7] and Musskopf et al. [8], who noted that the N1 system may yield less consistent stability in low-density bone.

The OS system, prepared at low speed without irrigation, yielded stability values comparable to CO. Previous investigations also reported that OS does not consistently outperform subtractive drilling in terms of mechanical stability [13,29,30]. It should be noted that, in this study, the OS protocol was tested only in combination with the N1 implant design. This lack of pairing with the parallel-walled implant may represent a methodological limitation, as it prevents the direct comparison of OS outcomes across different macrogeometries.

Thermal changes followed a different pattern. OD produced the highest ΔT, consistent with prior reports linking densification to increased frictional heat [31,32]. Nevertheless, all values remained below the critical threshold of 47 °C for osteonecrosis [11], confirming that adequate irrigation is sufficient to maintain thermal safety. OS, which was performed without irrigation, produced slightly higher ΔT than CO, consistent with earlier ex vivo observations [13,29,30]. However, the thermal assessment relied on a single-point infrared measurement at the crestal level. While this provided standardized and reproducible data, it may not have fully captured localized heating within deeper parts of the osteotomy.

**Figure 4:**
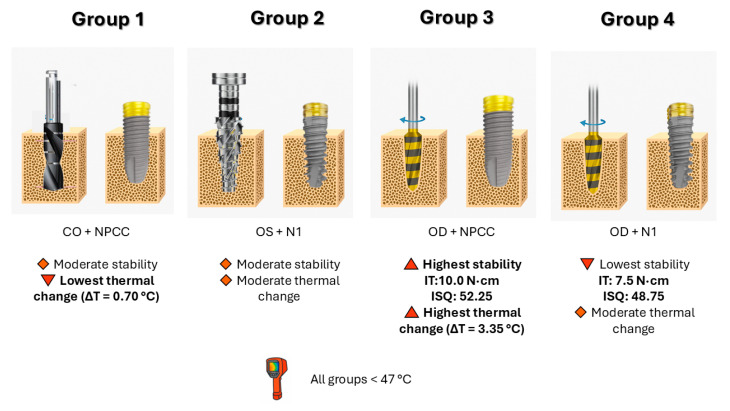
Schematic summary of the study outcomes and mechanism. Osseodensification with NPCC achieved the highest torque and stability but also the greatest heat rise, whereas OD + N1 showed the lowest stability. CO + NPCC resulted in minimal thermal increase with moderate stability, and OS + N1 demonstrated intermediate outcomes.

The overall outcomes and interactions between osteotomy techniques and implant macrogeometries are summarized schematically in Figure 4. To further interpret these findings, the nonlinear tendency observed among the study groups can be explained by the interaction between osteotomy-induced bone compaction, implant macrogeometry, and the resulting thermal–mechanical balance.

In this in vitro D4 bone model, osseodensification combined with the parallel-walled NPCC implant (OD + NPCC) achieved the highest insertion torque and ISQ values but also the greatest ΔT, indicating that strong trabecular compaction enhances primary stability at the expense of increased frictional heat. When the same densified cylindrical osteotomy was used with the tri-oval N1 implant (OD + N1), stability decreased despite comparable thermal generation, suggesting that the tri-oval geometry did not fully engage the densified walls, reducing circumferential stress transfer and mechanical interlocking. In contrast, the conventional osteotomy (CO + NPCC) removed bone chips and produced the lowest ΔT but moderate stability, while the OsseoShaper (OS + N1) provided intermediate values due to low-speed shaping with partial densification. These differences demonstrate that the nonlinear behaviour of IT, ISQ, and ΔT does not follow a simple linear correlation but reflects the interplay between compaction intensity, macrogeometry fit, and frictional work at the bone–implant interface. Such findings emphasize that optimal performance in low-density bone depends on matching the drilling protocol with the implant design to achieve both mechanical and thermal equilibrium.

Clinically, these results underscore the combined importance of osteotomy protocol and implant macrogeometry in D4 bone, where achieving predictable stability is especially challenging. OD with the NPCC implant may provide the most reliable outcomes, supporting its use in cases requiring immediate or early loading. Conversely, OD with the N1 (tri-oval design) implant appears to be less suitable for severely resorbed ridges. CO with the NPCC implant offered the lowest thermal rise while maintaining adequate stability, suggesting that this combination also remains a safe option.

These results are consistent with both recent experimental studies and classical observations. Jaffin and Berman [2] showed that implants in type IV bone are more likely to fail early, while Misch’s classification pointed out the clinical difficulties involved with this bone type [33]. Similarly, Friberg et al. [34] showed a link between bone density and initial stability in experimental models. In this context, the lower stability of OD + N1 may be explained by a biomechanical mismatch: osseodensification compacts bone into a cylindrical cavity, whereas the tri-oval N1 design reduces the contact area, decreasing the benefit of densification. By contrast, OD + NPCC gave the best balance of torque and stability, although with a higher thermal rise. Importantly, CO + NPCC, while not providing the highest stability, caused minimal thermal changes and achieved acceptable stability, suggesting it could be a safe clinical alternative when advanced drilling systems are not available.

Beyond stability, thermal control during osteotomy is another parameter that may influence peri-implant bone health. Excessive intraosseous heating has been reported as a potential contributor to delayed osseointegration and bone necrosis [11,35]. In this study, although osseodensification generated the highest thermal rise, all values remained well below the osteonecrosis threshold, indicating that, under standardized irrigation, innovative osteotomy techniques can maintain thermal safety. From a clinical perspective, this could suggest that when proper irrigation and appropriate implant macrogeometry are applied, both mechanical and biological safety may be achievable. Such approaches, if confirmed in future in vivo and clinical investigations, may contribute to reducing the incidence of early implant failure and peri-implant disease [36,37].

This study has several inherent limitations. First, the use of polyurethane foam blocks ensured standardization and reproducibility but excluded biological factors such as vascularity, bone remodeling, and healing. Second, only one implant size was tested, which may restrict the generalizability of the findings to other implant dimensions or clinical scenarios. Third, ISQ measurements reflected only primary stability and did not provide information about long-term osseointegration. Fourth, the OsseoShaper protocol was tested exclusively with the tri-oval N1 implant, limiting the ability to compare its performance across different macrogeometries. Finally, thermal analysis was performed using single-point infrared thermography at the crestal level. Although this method is standardized and reproducible, it may not fully capture localized thermal changes in deeper or lateral regions of the osteotomy site. These clinical implications should therefore be interpreted with caution, as the present study was performed on synthetic bone models and did not account for biological healing responses.

The present findings may carry important clinical implications for reducing the risk of implant failure in low-density bone. Previous reports have consistently shown that poor primary stability is a critical factor underlying early implant loss, particularly in type IV bone [2,34]. Micromotion at the bone–implant interface has also been identified as a factor that could compromise osseointegration and predispose implants to failure [38,39]. Within the limitations of this in vitro study, the observation that osseodensification combined with a parallel-walled macrogeometry yielded higher insertion torque and ISQ values suggests that this approach might enhance the predictability of osseointegration and lower the likelihood of early complications. Conversely, the combination of osseodensification with a tri-oval macrogeometry showed reduced stability, which could be associated with a higher risk of early failures in clinical conditions. These results highlight the potential importance of carefully matching osteotomy protocols with implant design to mitigate risks in challenging scenarios.

## 5. Conclusions

Within the limitations of this in vitro study, OD + NPCC provided the best combination of torque and stability but with a higher thermal increase, while CO + NPCC offered a thermally safe and clinically acceptable alternative. These findings highlight the clinical importance of matching osteotomy techniques with implant design in low-density bone. Further in vivo studies are warranted to confirm these results and strengthen their clinical relevance.

## Figures and Tables

**Table 1:** Study group design, osteotomy protocols, and implant types.

Group	Osteotomy Technique	Implant Design
1	CO	NPCC (4.3 × 11.5 mm)
2	OS	N1 (4.0 × 11 mm)
3	OD	NPCC (4.3 × 11.5 mm)
4	OD	N1 (4.0 × 11 mm)

Description of the experimental groups, including osteotomy technique and implant designs. CO = conventional osteotomy; OS = OsseoShaper; OD = osseodensification.

**Table 2:** Summary of IT, ΔT, and ISQ (median with range).

Group	IT Median (N·cm) (Min–Max)	ΔT Median (°C) (Min–Max)	ISQ Median (Min–Max)
Group 1	8.0 (5.0–10.0)	0.70 (0.40–3.30)	52.5 (44.0–58.5)
Group 2	8.0 (7.0–10.0)	1.75 (0.60–4.10)	50.0 (49.5–59.0)
Group 3	10.0 (8.0–12.0)	3.35 (1.30–4.80)	52.25 (50.0–53.5)
Group 4	7.5 (7.0–8.0)	2.60 (2.20–3.70)	48.75 (47.0–49.5)
*p* values	0.001	0.001	0.001

Median values with full ranges (minimum–maximum) are presented for insertion torque (IT), thermal change (ΔT), and implant stability quotient (ISQ), providing a clear representation of central tendency and variability across groups. All outcomes showed statistically significant group differences (Kruskal–Wallis test, *p* = 0.001).

**Table 3:** Pairwise comparisons.

Comparison	IT (Ncm)	ΔT (°C)	ISQ
Group 1/Group 3	**0.040**	**0.004**	1.000
Group 2/Group 4	0.090	1.000	**0.002**
Group 1/Group 4	1.000	0.290	**0.040**
Group 2/Group 3	0.160	**0.010**	**0.010**
Group 1/Group 2	1.000	**0.040**	0.120
Group 3/Group 4	**0.002**	0.720	**0.001**

Pairwise comparisons between groups for IT, ΔT, and ISQ using Dunn–Bonferroni adjusted *p*-values. Values in bold indicate statistically significant differences (*p* < 0.05).

## Data Availability

The datasets generated and analyzed during the current study are available from the corresponding author on reasonable request.

## Supplementary Materials

The following supporting information can be downloaded at https://www.mdpi.com/article/10.3390/bioengineering12111155/s1. Figure S1: Representative images of osteotomy site preparations performed in polyurethane foam blocks. The figure illustrates the characteristic appearance of the osteotomies created with the CO, OS, and OD techniques before implant placement. Figure S2: Evaluation of primary stability by resonance frequency analysis (RFA). After implant placement, MultiPeg abutments were hand-tightened, and ISQ values were measured in buccolingual and mesiodistal directions using the Penguin RFA device. The mean of the two readings was used for analysis. Figure S3: Temperature changes (ΔT) were recorded during osteotomy with an infrared thermal camera (UNI-T UTI720E) positioned 25 cm from the site. A single-point measurement was taken at the alveolar crest, and values were extracted frame by frame. The setup ensured stable positioning and reproducible alignment throughout all recordings.

## Abbreviations

The following abbreviations are used in this manuscript:

CO	Conventional Osteotomy
OD	Osseodensification
OS	OsseoShaper
NPCC	NobelParallel Conical Connection
N1	Nobel Biocare N1
IT	Insertion Torque
ISQ	Implant Stability Quotient
ΔT	Thermal Change
RFA	Resonance Frequency Analysis
SD	Standard Deviation
ICC	Intraclass Correlation Coefficient
ASTM	American Society for Testing and Materials
