# Supplementary material for: Thermal and Stability Outcomes of Different Osteotomy Techniques and Implant Macrogeometries in Type IV Bone: An In Vitro Study
Source: Bioengineering (Basel). 2025 Oct 24;12(11):1155. doi: 10.3390/bioengineering12111155 (PMC12649583; doi:10.3390/bioengineering12111155)
Supplement: Supplementary file 1 [file bioengineering-12-01155-s001.zip › bioengineering-3916622-supplementary/Supplementary Materials Footnotes.pdf]

Figure Footnotes for Supplementary Materials

Figure S1: Representative images of osteotomy site preparations performed in polyurethane foam blocks. The figure illustrates the characteristic appearance of the osteotomies created with the CO, OS, and OD techniques before implant placement.

Figure S2: Evaluation of primary stability by resonance frequency analysis (RFA). After implant placement, MultiPeg abutments were hand-tightened, and ISQ values were measured in buccolingual and mesiodistal directions using the Penguin RFA device. The mean of the two readings was used for analysis.

Figure S3: Temperature changes (ΔT) were recorded during osteotomy with an infrared thermal camera (UNI-T UTI720E) positioned 25 cm from the site. A single-point measurement was taken at the alveolar crest, and values were extracted frame by frame. The setup ensured stable positioning and reproducible alignment throughout all recordings.
