# Supplementary figures and images for: Thermal and Stability Outcomes of Different Osteotomy Techniques and Implant Macrogeometries in Type IV Bone: An In Vitro Study
Source: Bioengineering (Basel). 2025 Oct 24;12(11):1155. doi: 10.3390/bioengineering12111155 (PMC12649583; doi:10.3390/bioengineering12111155)

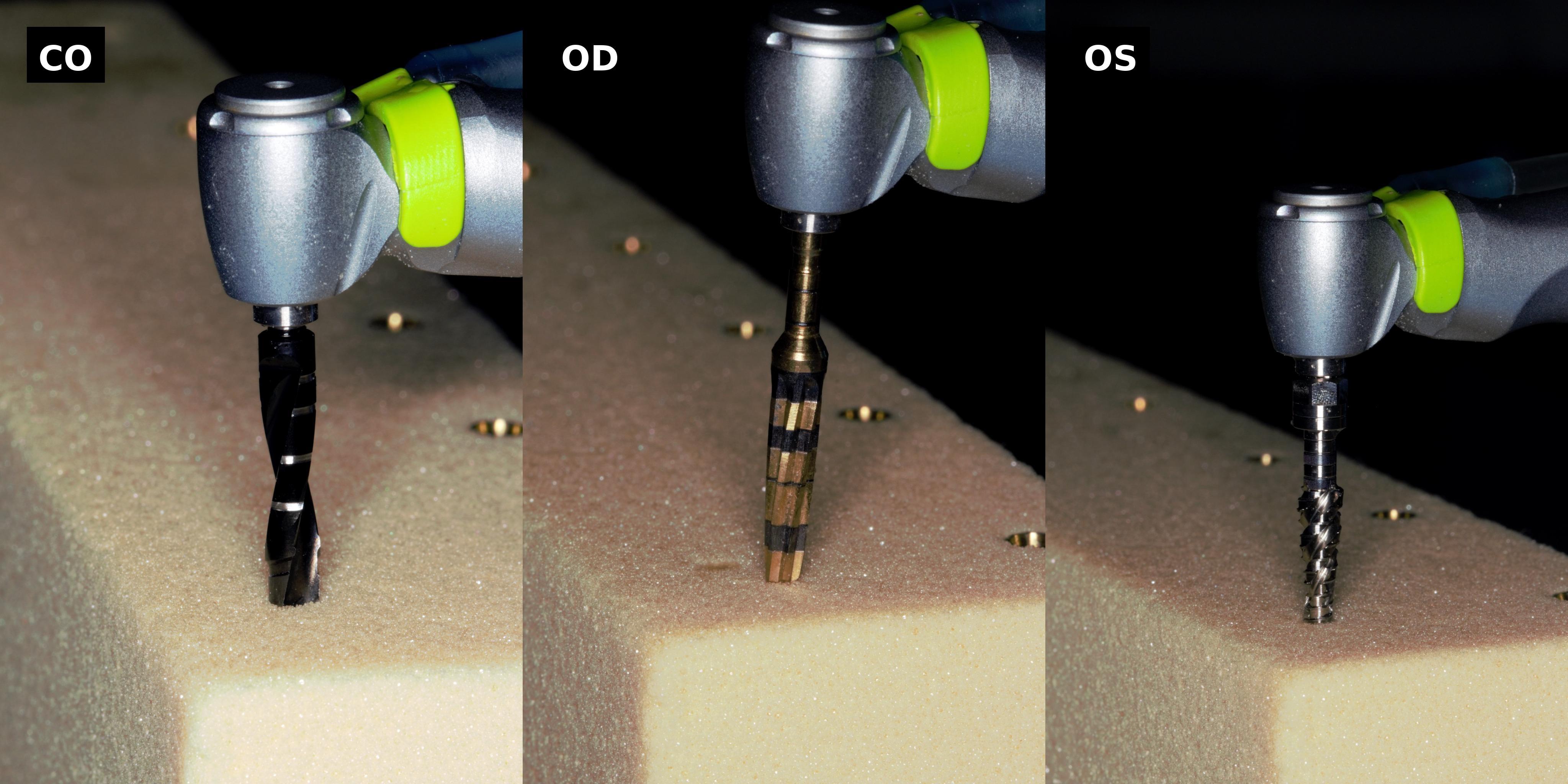

Supplement: Supplementary file 1 [file bioengineering-12-01155-s001.zip › bioengineering-3916622-supplementary/Figure s1.jpg]

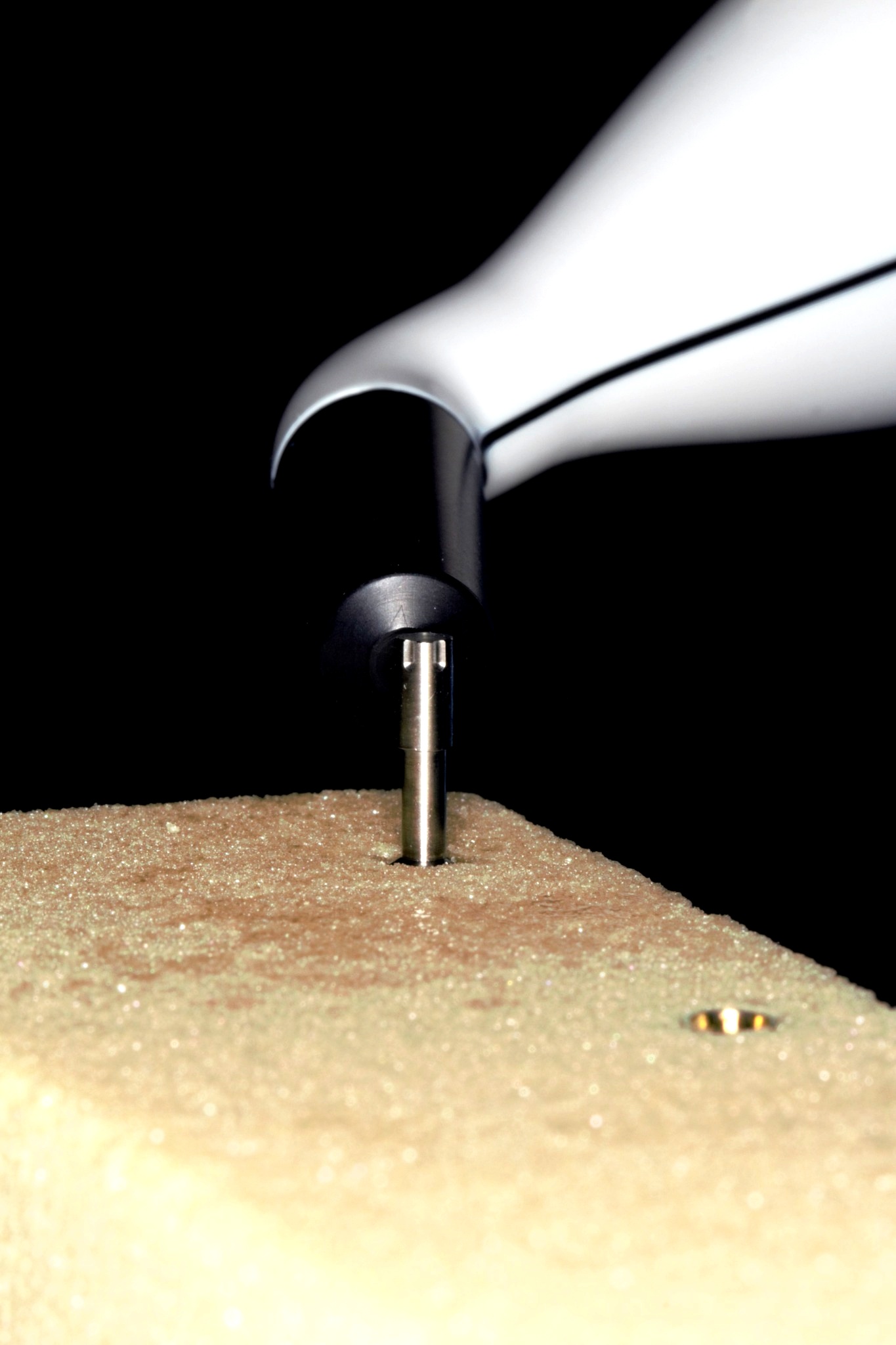

Supplement: Supplementary file 1 [file bioengineering-12-01155-s001.zip › bioengineering-3916622-supplementary/Figure s2.jpg]

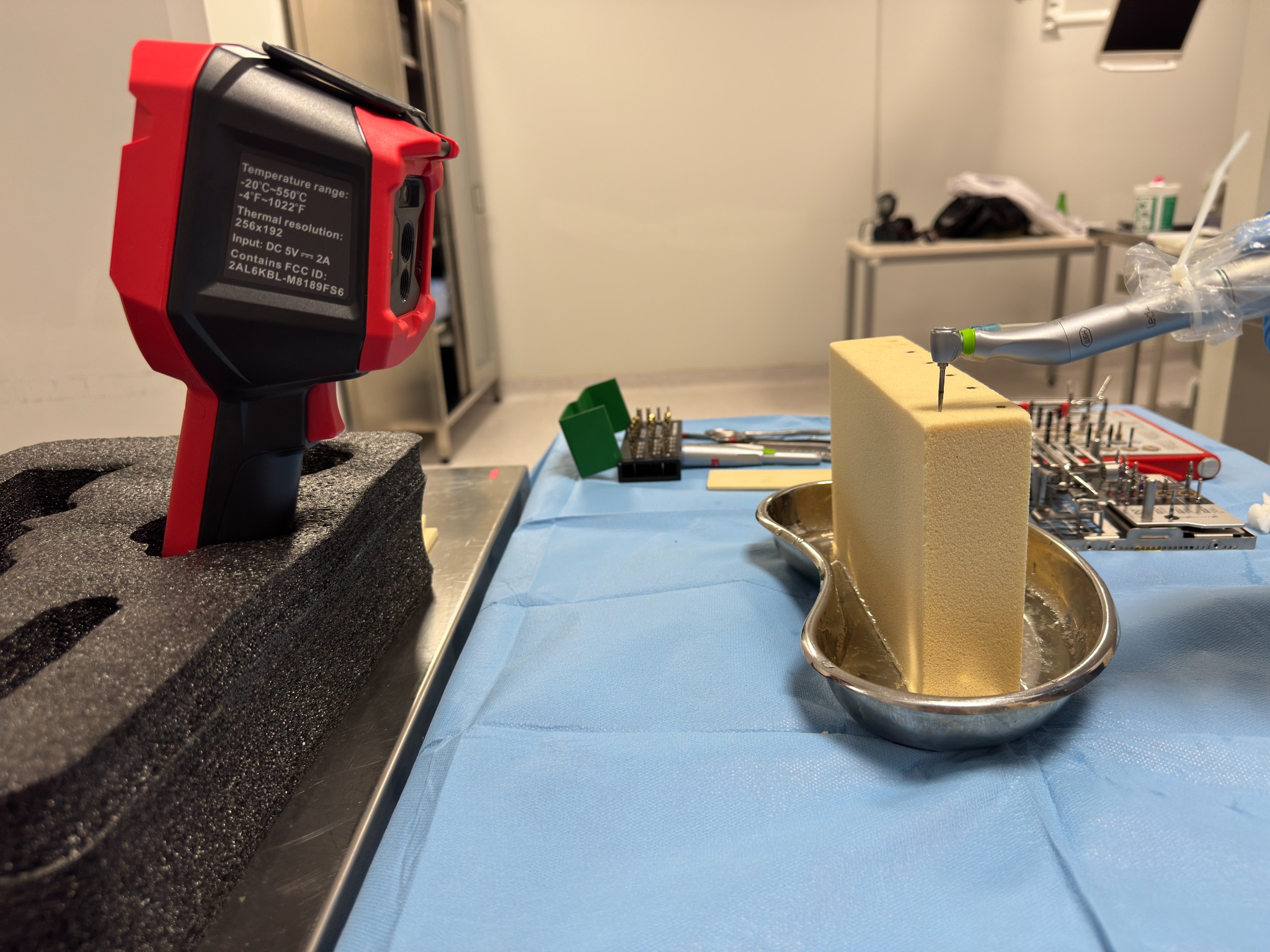

Supplement: Supplementary file 1 [file bioengineering-12-01155-s001.zip › bioengineering-3916622-supplementary/figure s3.JPEG]
